# Elevated Expression of Lumican in Lung Cancer Cells Promotes Bone Metastasis through an Autocrine Regulatory Mechanism

**DOI:** 10.3390/cancers12010233

**Published:** 2020-01-17

**Authors:** Kuan-Chung Hsiao, Pei-Yi Chu, Gee-Chen Chang, Ko-Jiunn Liu

**Affiliations:** 1 National Institute of Cancer Research, National Health Research Institutes, Tainan 70456, Taiwan; randolph.hsiao@gmail.com; 2 Department of Pathology, Show Chwan Memorial Hospital, Changhua 50008, Taiwan; chu.peiyi@msa.hinet.net; 3 School of Medicine, College of Medicine, Fu Jen Catholic University, New Taipei 24205, Taiwan; 4 Faculty of Medicine, School of Medicine, National Yang-Ming University, Taipei 11266, Taiwan; 5 Division of Chest Medicine, Department of Internal Medicine, Taichung Veterans General Hospital, Taichung 40764, Taiwan; 6 Institute of Biomedical Sciences, National Chung-Hsing University, Taichung 40227, Taiwan; 7 Institute of Clinical Pharmacy and Pharmaceutical Sciences, National Cheng Kung University, Tainan 70101, Taiwan; 8 School of Medical Laboratory Science and Biotechnology, Taipei Medical University, Taipei 11041, Taiwan

**Keywords:** bone metastasis, lumican, lung cancer

## Abstract

*Background*: The microarray analysis of whole-genome expression indicated that the gene encoding the protein lumican, which is associated with extracellular matrix (ECM) interaction, was highly expressed in osteotropic lung cancer cell lines with an enhanced capacity of bone metastasis. *Methods*: The expression of lumican in the osteotropic lung cancer cells was downregulated, and the in vitro migration, invasion, and adhesion of cancer cells to ECM components, and the in vivo bone metastasis capacity of these cells were examined. Exogenous lumican was provided to study the autocrine regulation mechanism of lumican in the bone metastasis of lung cancer cells. *Results*: Transfection with lumican-specific short hairpin RNA (shRNA) in the osteotropic lung cancer cells reduced the establishment of in vivo bone metastasis, but not lung metastasis. Reduction in the expression of lumican also decreased the attachment of lung osteotropic cancer cells to several components of the ECM and suppressed cell migration and invasion in vitro. Exogenous lumican restored these reduced capacities of lumican knockdown cells and promoted the seeding of lung cancer cells in the bone microenvironment. *Conclusions*: These results suggested that lumican promotes the metastasis of lung cancer cells to the bones via an autocrine regulatory mechanism, and blocking this interaction may provide a new therapeutic approach to reduce bone metastasis in cases of lung cancer.

## 1. Introduction

Tumor metastasis is a complex process involving multiple steps and is the major cause of cancer-related deaths in humans [1]. Multiple-organ metastasis is usually observed in patients with lung cancer of advanced stages [2]. In lung cancer, bone is one of the common metastatic sites, and 30% to 40% of non-small-cell lung carcinoma (NSCLC) patients exhibit bone metastasis at the time of initial staging [3,4]. The prominent sites of bone metastases include the axial skeleton and proximal long bones such as the spine, ribs, and ilium [5]. Pain in the bones is frequently observed in 80% of patients with lung cancer showing bone metastases, and their median survival time is less than six months [6,7]. Therefore, reducing the incidence of bone metastasis may provide significant clinical benefits for patients with lung cancer.

Similar to the processes of metastases in other tumors, to cause tumor metastasis in the bone, the following steps occur in the body: Cells escape from their primary site and enter circulation; they adhere to the bone matrix and get arrested in the bone marrow; finally, the cells proliferate and colonize in the bone marrow [8]. Due to the unique composition of the bone [9], we hypothesized that tumor cells might be undergoing several genetic alterations during the course of tumor progression, and promotion of cell growth in the bone microenvironment might be occurring due to enhanced interaction between the tumor cells, bone stromal cells, bone extracellular matrix (ECM), osteoblasts, and osteoclasts. We believe that understanding the molecular mechanism underlying bone metastasis may help us to develop a molecular therapy that could be targeted toward the bone metastasis of lung cancer.

To evaluate the molecular mechanism underlying bone metastasis associated with lung cancer, in this study, we used a murine lung cancer cell line, *luciferase*-transfected Lewis lung carcinoma (LLC/luc), to isolate osteotropic cancer cells from the bone by in vivo selection. After analyzing the transcriptomic profiles of parental and osteotropic cells, a gene encoding the protein lumican, which is associated with ECM interaction, was identified, and the expression of this gene was elevated in the osteotropic LLC/luc cells compared to the parental cells.

Lumican is a protein belonging to the small family of leucin-rich proteoglycans and is a major proteoglycan component of the bone matrix that is mainly expressed in osteoblasts during the differentiation and maturation stages, but not in the proliferation stage [10]. Early study demonstrated an important role of lumican in collagen fibrillogenesis [11]. Lumican is known to be expressed in several cancer types including breast, pancreatic, and colon cancers [12,13,14]. An increased expression of lumican was observed in breast-infiltrating ductal carcinoma, as compared to that in normal breast tissue [15]. Overexpression of lumican in human colon cancer cells increased its secretion and enhanced the migration ability of cancer cells through remodeling the rearrangement of actin cytoskeleton and the localization of gelsolin in cytoplasm [16]. In addition, colorectal cancer patients with a high expression level of lumican had poor prognosis as compared to those with a low expression level of lumican [17]. Lumican was found to promote the migration of neutrophils by depositing itself on the cell surface and interacting with the β2 integrins. It also plays a role in the extravasation of neutrophils following corneal injury and wound healing [18,19]. Another study showed that lumican induced cell migration and proliferation via the ERK1/2 signaling pathway in human corneal epithelial cells [20]. In the human lung cancer cell line, A549, lumican was found to promote cell proliferation by increasing the expression of Ras homolog gene family, member C (RhoC) and phosphorylated protein kinase B (p-Akt) [21]. Additionally, it was reported that lumican regulates the downstream Smad2/3 (homologies to the SMA in *Caenorhabditis elegans* and MAD in *Drosophila*) signaling of the transforming growth factor beta (TGF-β) signaling pathway that controls bone formation [22]. These studies suggest that lumican is involved in the bone metastasis of lung cancer cells by modulating bone remodeling, increasing the mobility, and serving as a chemoattractant for lung cancer cells, thereby promoting cell invasion. In this study, we aimed to investigate the role of lumican in several steps of the metastasis process and evaluate its effect on bone homeostasis.

## 2. Results

**Figure 1:**
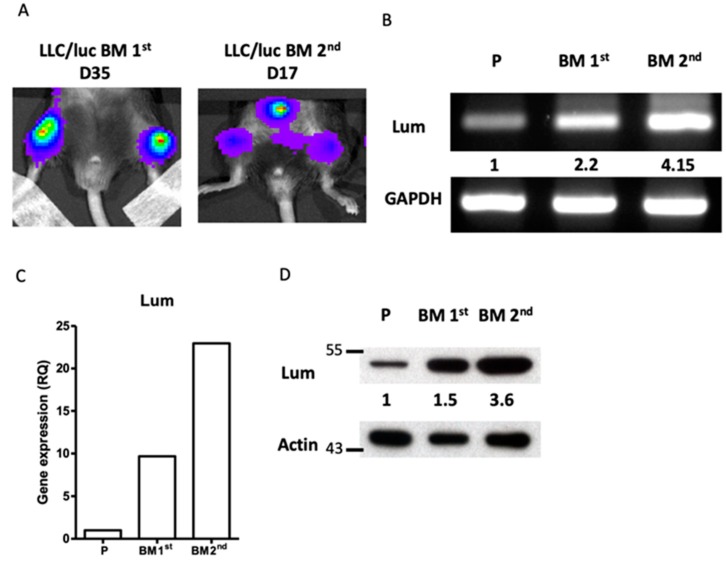
Expression of lumican in osteotropic LLC/luc cells. (**A**) To develop lung cancer cells with a higher capacity of bone metastasis, a mouse LLC lung cancer cell line transfected with the *luciferase* gene (LLC/luc) was injected into the left ventricle of a C57BL/6 mouse. After 35 days (D35), the luciferase activity was observed in the femurs of mice by the in vivo imaging system(IVIS). The bone marrow cells of mice with bone metastases were collected and cultured in vitro to establish the first bone metastatic cell line, LLC/luc BM 1st. The BM 1st cells were injected again into a different mouse and the luciferase activity was detected on D17. The bone marrow cells of this mouse were collected and cultured in vitro to establish a second cell line exhibiting high bone metastasis, LLC/luc BM 2nd. The expression of lumican in the parental LLC/luc (P), LLC/luc BM 1st, and LLC/luc BM 2nd cells was determined by RT-PCR (**B**), quantitative-real-time PCR (**C**), and Western blot analysis (**D**). The level of lumican expression in each cell was individually normalized to the internal control (GAPDH or actin), and the numbers in (**B**,**D**) indicate the expression levels of lumican in the bone metastatic LLC/luc cells as compared to those in the parental LLC/luc cells (level set to 1).

Lumican expression was increased in the LLC/luc cells with an enhanced capacity of bone metastasis. Initially, we adopted a mouse model to study the genes involved in the bone metastasis of lung cancer. A mouse lung cancer cell line, LLC, transfected with the *luciferase* gene (LLC/luc), was injected intracardially (I.C.) into the left ventricle of mice as described previously [23]. Metastasis of LLC/luc cells in the bones of mice was observed after 35 days of injection (Figure 1A, left panel). Cancer cells were harvested from the metastatic lesions in the tibia and cultured in vitro to establish the first bone metastatic cancer cell sub-line, LLC/luc BM 1st. The LLC/luc BM 1st cells were re-injected into the mice I.C., and a faster development of bone metastasis, 17 days after the injection, was observed (Figure 1A, right panel). Cancer cells harvested from the metastatic lesions were cultured to develop the secondary bone metastatic cancer cell sub-line, LLC/luc BM 2nd. To identify the genes involved in bone metastasis, we compared the global gene expression profiles of the parental LLC/luc, LLC/luc BM 1st, and LLC/luc BM 2nd cells using microarray analysis. The significant, differentially expressed genes were identified as |log2 (genes expressed in LLC BM 2nd/genes expressed in LLC P)| > 1, and we found that the expression of the gene encoding for lumican, *LUM*, was the highest among the 269 upregulated genes in LLC BM 2nd cells (Table S1). Lumican is known to play an important role in the remodeling of ECM. Owing to the importance of the regulation of ECM remodeling and cell adhesion in cancer metastasis, we examined genes involved in these pathways by using the Database for Annotation, Visualization, and Integrated Discovery (DAVID) online tool. The functional annotation results showed that several genes associated with ECM and epithelial-mesenchymal transition (EMT) were enriched in our model (Table S2). To verify the microarray results, we used RT-PCR, Q-RT-PCR, and Western blotting to analyze the levels of lumican expression in the parental, LLC/luc BM 1st, and LLC/luc BM 2nd cells. As shown in Figure 1B–D and Figure S4 (Supplementary Materials), the expression of lumican in LLC/luc BM 2nd cells (hereafter referred to as the osteotropic LLC/luc cells) was higher than that in the parental and LLC/luc BM 1st cells at both messenger RNA (mRNA) and protein levels.

**Figure 2:**
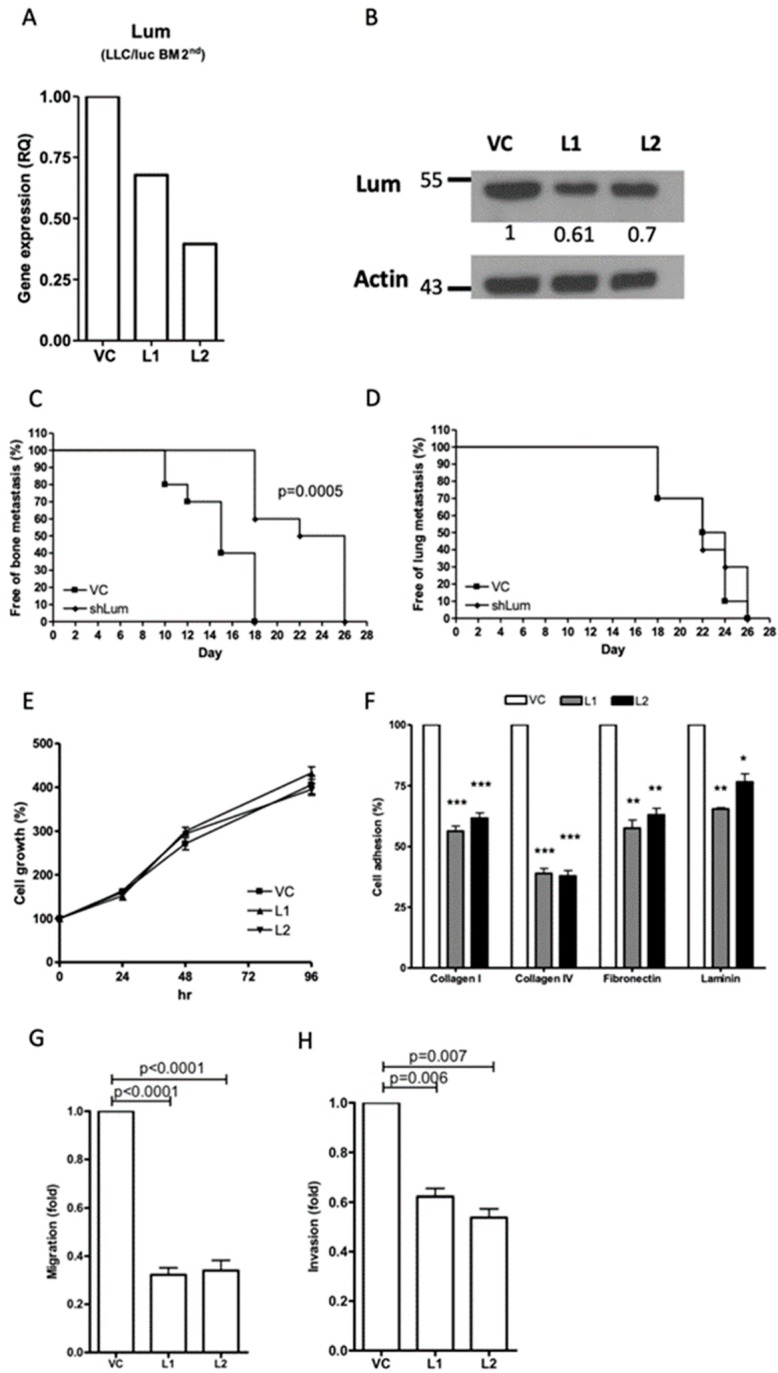
Effect of lumican knockdown on the function of bone metastatic LLC/luc BM 2nd cells. The expression of lumican in LLC/luc BM 2nd cells transfected with a control vector (VC) and a lumican-specific short hairpin RNA (shRNA) plasmid (L1 and L2) was determined by real-time RT-PCR (**A**) and Western blot analysis (**B**). The level of lumican expression in each cell was individually normalized to the internal control (actin), and the numbers in (**B**) indicate the level of lumican expression in lumican knockdown LLC/luc BM 2nd cells as compared to that in the cells transfected with the control vector. The LLC/luc BM 2nd cells transfected with a control vector (VC) and a lumican-specific shRNA (shLum) were administered by injecting them intracardiac (I.C.) and intravenous (I.V.) to allow the establishment of bone (**C**) and lung (**D**) metastasis (*n* = 10, from two separate experiments), respectively. The presence of tumor metastasis as determined by the presence of luciferase activity was detected by the IVIS imaging system. The cell proliferation (**E**) and adhesion to the extracellular matrix (ECM) components (**F**) of LLC/luc BM 2nd cells transfected with the control vector (VC) and the lumican-specific shRNA plasmid (L1, L2) were determined by an Methylthiazolyldiphenyl-tetrazolium bromide (MTT) assay and a cell adhesion assay, respectively. The migration (**G**) and invasion (**H**) abilities of LLC/luc BM 2nd cells transfected with the control vector and the lumican-specific shRNA plasmid were determined by the Transwell migration assay. * *p* ≤ 0.05, ** *p* ≤ 0.01, and *** *p* ≤ 0.001. The error bars are defined as means ± SEM.

Downregulation of lumican reduced the capacity for bone metastasis, but not lung metastasis, in the LLC/luc BM 2nd cells. To directly examine the role of lumican in tumor metastasis, we transfected a lumican-specific short hairpin RNA (shRNA) vector into the bone metastatic LLC/luc BM 2nd cells. The expression of lumican in two separate lumican knockdown cell lines was decreased for mRNA and protein levels (Figure 2A,B and Figure S5, Supplementary Materials) as compared to that of cells transfected with a control vector. Subsequently, the LLC/luc BM 2nd cells transfected with a control vector and a lumican-specific shRNA vector were injected I.C. and intravenously (I.V.) into mice to evaluate the development of bone and lung metastases, respectively. As shown in Figure 2C,D, lumican downregulation in the LLC/luc BM 2nd cells delayed the development of bone metastasis, but it had no influence on the lung metastasis under this experimental setting.

Downregulation of lumican expression suppressed cell migration and invasion and decreased cell adhesion to ECM components. To investigate the potential mechanisms involved in the promotion of bone metastasis mediated by lumican, the growth of lumican knockdown LLC/luc BM 2nd cells was analyzed in vitro. Although the effect of lumican on cell proliferation was reported [13,24], we found that the proliferation rate of lumican knockdown LLC/luc BM 2nd cells was the same as that of the cells transfected with a control vector (Figure 2E) suggesting that the concentration of lumican has no impact on the growth of LLC/luc cells in vitro. Previous studies also demonstrated that the expression of lumican is correlated with the ability of cell adhesion and cellular mobility [25,26]. Therefore, cellular adhesion, migration, and invasion analyses were performed. As shown in Figure 2F, adhesion of the LLC/luc BM 2nd cells to several ECM components including collagen I, collagen IV, fibronectin, and laminin was significantly decreased in the lumican knockdown cells. Moreover, both cell migration and invasion abilities were also reduced after lumican downregulation (Figure 2G,H). These results suggest that lumican participates in the adhesion of cancer cells to the ECM components, followed by migration and invasion, which are critical steps in the establishment of tumor metastasis.

**Figure 3:**
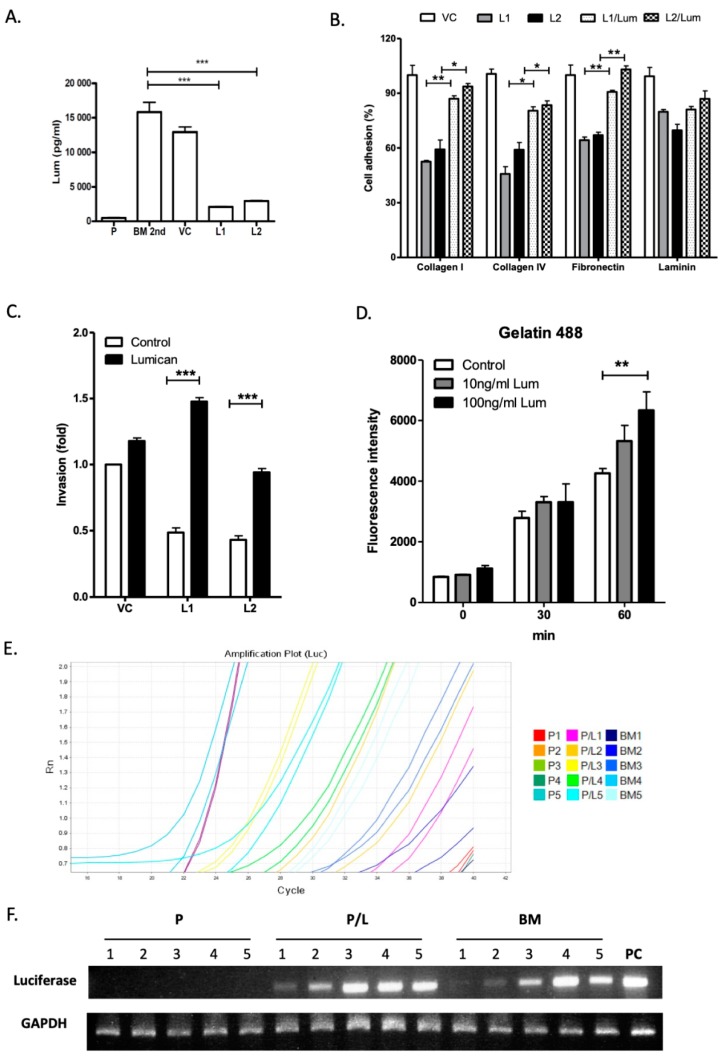
Effect of exogenous lumican on function of lumican knockdown bone metastatic LLC/luc cells. (**A**) The level of lumican in the culture supernatant of the parental LLC/luc (P), LLC/luc BM 2nd (BM 2nd), and LLC/luc BM 2nd cells transfected with a control and a lumican-specific shRNA vector was determined by an ELISA assay. (**B**) Cell adhesion to different ECM components was determined in the bone metastatic cells transfected with a control vector, LLC/luc cells (VC), and lumican knockdown LLC/luc BM 2nd cells incubated with (L1/lum and L2/lum) and without (L1 and L2) the recombinant lumican protein. (**C**) Cell invasion was determined in LLC/luc BM 2nd cells transfected with a control (VC) and a lumican-specific shRNA vector (L1 and L2) after incubation with (lumican) and without (control) the recombinant lumican protein, respectively. (**D**) Lumican knockdown LLC/luc BM 2nd cells were cultured in a medium containing the recombinant lumican protein (10 ng/mL and 100 ng/mL) at 37 °C. In total, 100 μL of the culture supernatant was collected after intervals of 30 min and incubated with fluorescein-conjugated gelatin as a substrate for matrix metalloproteinase 2/9 (MMP2/9). The fluorescence released from the degraded substrates was then measured. (**E**) The LLC/luc parental cells co-cultured with (P/L1 to P/L5) and without (P1 to P5) recombinant lumican protein and LLC/luc BM 2nd cells (BM1 to BM5) were injected into mice (*n* = 5) I.C. The total RNA of bone marrow cells was extracted one day after injecting them I.C. The expression of luciferase gene was determined by quantitative-real-time PCR to detect the presence of tumor cells in the bone marrow, and the amplification of luciferase gene was confirmed by agarose gel electrophoresis (**F**). GAPDH was used as an internal control. The DNA extracted from the parental LLC/luc cells was used as a positive control (PC). * *p* ≤ 0.05, ** *p* ≤ 0.01, and *** *p* ≤ 0.001.

Exogenous lumican restores cell adhesion and invasion capacities in the lumican knockdown LLC/luc BM 2nd cells. It was reported that binding of lumican to surface integrins promotes the migration of neutrophils [18,27]. To investigate the autocrine and paracrine effects of lumican on cancer cell mobility, the secretion of lumican in the parental LLC/luc, LLC/luc BM 2nd, and lumican knockdown LLC/luc BM 2nd cells was analyzed. As shown in Figure 3A, the secretion of lumican in the LLC/luc BM 2nd cells was significantly higher than its secretion in the parental cells, and the amount of lumican decreased significantly in lumican knockdown LLC/luc BM 2nd cells. Because surface expression of the integrins β1 and β2, which are known to be receptors of lumican [27], was detected in the LLC/luc BM 2nd cells (Figure S1, Supplementary Materials), it is likely that lumican promotes the invasive and metastatic abilities of LLC/luc BM 2nd cells via an autocrine signaling process. To further study the role of extracellular lumican in the interaction of ECM components and lung cancer cells, the LLC/luc BM 2nd cells transfected with a lumican-specific shRNA and control vectors were cultured with and without the recombinant mouse lumican protein, and their cell adhesion and invasion capacities were determined. As shown in Figure 3B, decreased adhesion of the lumican knockdown LLC/luc BM 2nd cells to collagen I/IV and fibronectin was restored with the addition of exogenous lumican, but reduced adhesion to laminin was not recovered. Nevertheless, the reduced ability of invasion in the lumican knockdown cells was also restored in the presence of exogenous lumican (Figure 3C).

Lumican modulates focal adhesion kinase (FAK) signaling, proteolytic activity, and early metastasis seeding of LLC/luc BM 2nd cells. Lumican was shown to affect cell functions by binding to α2β1 integrins, resulting in FAK signal activation [20,27,28]. We found that knocking down lumican suppressed FAK phosphorylation in the LLC/luc BM 2nd cells, which was reverted with the addition of exogenous lumican (Figure S2, Supplementary Materials). FAK signaling plays a critical role in the production of matrix metalloproteinase (MMP)2 or MMP9 in tumor cells [29]. The increased ability of invasion in the LLC/luc cells treated with exogenous lumican suggests that the proteolytic activity associated with ECM degradation in the LLC/luc cells is elevated after lumican stimulation. Therefore, we determined the protease activity in lumican knockdown LLC/luc BM 2nd cells co-cultured with exogenous lumican by using fluorescein-conjugated gelatin as a substrate. As shown in Figure 3D, increased protease activity in the cells co-cultured with a higher dose of exogenous lumican was observed after 60 min.

Next, we examined whether the autocrine regulation of lumican results in an enhanced seeding of the LLC/luc BM 2nd cells in the bone microenvironment. As shown in Figure 3E,F, the presence of LLC/luc BM 2nd cells, but not the parental LLC/luc cells, was detected in the bone marrow one day after injecting the mice I.C. Co-culture of the parental LLC/luc cells with lumican also resulted in the detection of cancer cells one day after injecting them I.C. These results suggest that the seeding of parental LLC/luc cells into the bone microenvironment was enhanced after co-culture with exogenous lumican. Hence, the results demonstrated in this study indicated that lumican promotes tumor cell adhesion and invasion, as well as the incidence of bone metastasis of tumors, via an autocrine regulatory mechanism.

**Figure 4:**
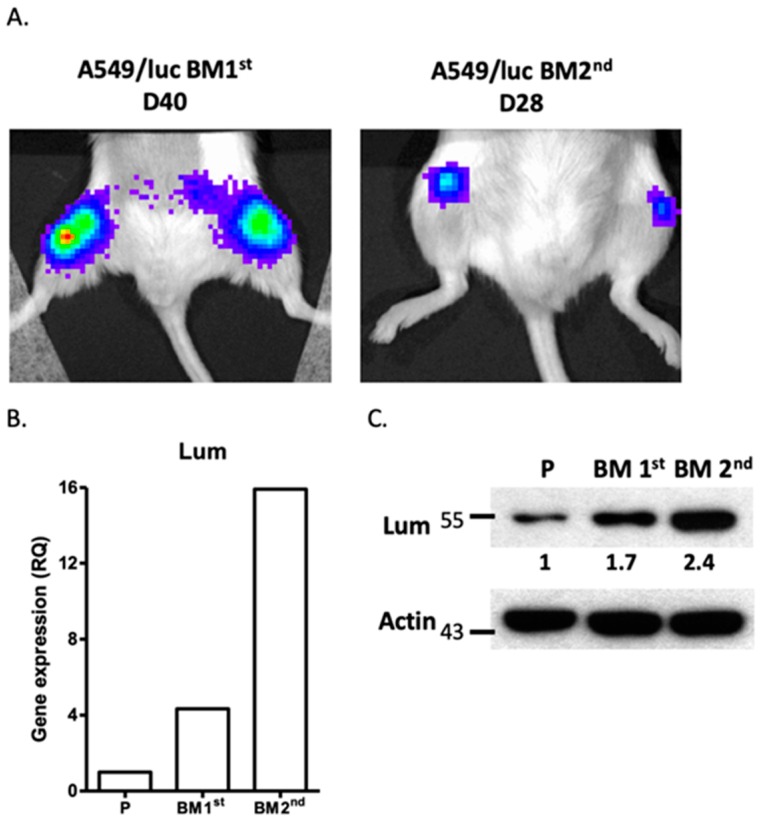
Expression of lumican in bone metastatic A549/luc cells. (**A**) The A549 human lung cancer cell line transfected with the *luciferase* gene (A549/luc) was injected into the left ventricle of a NOD-SCID mouse. After 40 days (D40), the luciferase activity was observed in the femurs of mice by the IVIS imaging system. The bone marrow cells of mice with bone metastasis were collected and cultured in vitro to establish the bone metastatic cell line, A549/luc BM 1st. The BM 1st cells were injected again into a different mouse and the luciferase activity was detected on D28. The bone marrow cells of this mouse were collected and cultured in vitro to establish a highly bone metastatic cell line, A549/luc BM 2nd. The expression of lumican in the parental A549/luc (P), A549/luc BM 1st, and A549/luc BM 2nd cells was determined by quantitative-real-time PCR (**B**) and Western blot analysis (**C**). The level of lumican expression in each cell line was individually normalized to the internal control (actin), and the numbers in (**C**) indicate the level of lumican expression in bone metastatic A549/luc cells as compared to the parental A549/luc cells (level set to 1).

Downregulation of lumican also decreased cell mobility and bone metastasis of the tumors in human A549 lung cancer cells. To extend our findings with mouse lung cancer cells to human cells, a human A549 lung cancer cell line transfected with the *luciferase* gene, A549/luc, was subjected to in vivo selection to determine its capacity for bone metastasis, as described above. Initially, bone metastasis was observed after 40 days of I.C. administration of the parental A549/luc cells into NOD-SCID mice, while the A549/luc BM 1st cells resulted in bone metastasis only 28 days after administration I.C. (Figure 4A). A highly metastatic A549 sub-line, A549/luc BM 2nd, was established. We found that the lumican expression was also upregulated in the A549/luc BM 1st and BM 2nd cells for both mRNA and protein levels (Figure 4B,C and Figure S6, Supplementary Materials).

**Figure 5:**
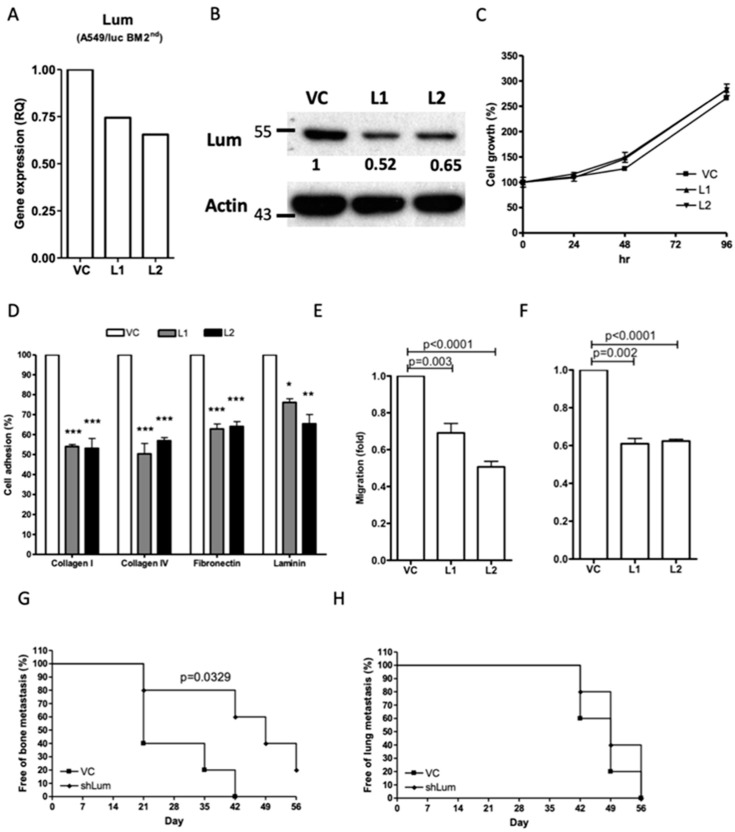
Effect of lumican knockdown on cellular function of bone metastatic A549/luc cells. The expression of lumican in bone metastatic A549/luc BM 2nd cells transfected with a control (VC) and a lumican-specific shRNA vector (L1 and L2) was determined by real-time RT-PCR (**A**) and Western blot analysis (**B**). The level of lumican expression in each cell was individually normalized to the internal control (actin), and the numbers indicate the level of lumican expression in lumican knockdown A549/luc BM 2nd cells as compared to that in the cells transfected with the control vector. The cell growth (**C**) and ECM component adhesion (**D**) of A549/luc BM 2nd cells transfected with a control and a lumican-specific shRNA vector was determined by an MTT assay and a cell adhesion assay. The migration (**E**) and invasion (**F**) abilities of A549/luc BM 2nd cells transfected with the control and the lumican-specific shRNA vector were determined by the Transwell migration assay. The A549/luc BM 2nd cells transfected with a control (VC) and a lumican-specific shRNA (shLum) vector were administered by intracardiac and intravenous injection to allow the establishment of the bone (**G**) and lung (**H**) metastasis (*n* = 5), respectively. The presence of tumor metastasis, as determined by the presence of luciferase activity, was detected by the IVIS imaging system. * *p* ≤ 0.05, ** *p* ≤ 0.01, and *** *p* ≤ 0.001. The error bars are defined as means ± SEM.

Transfection with a human lumican-specific shRNA vector resulted in a reduced mRNA and protein expression of lumican in the A549/luc BM 2nd cells (Figure 5A,B and Figure S7, Supplementary Materials) without influence on the cell proliferation (Figure 5C). Nevertheless, the cell adhesion, migration, and invasion abilities were all significantly inhibited in the A549/luc BM 2nd cells with lumican downregulation (Figure 5D–F). Additionally, bone metastasis of the tumor, but not lung metastasis, was delayed in the NOD-SCID mice injected with the A549/luc BM 2nd cells after lumican downregulation (Figure 5G,H). Taken together, these results indicated that lumican plays a specific role in bone metastasis in both mouse and human lung cancer cells.

## 3. Discussion

The focus of this study was to evaluate the role of lumican in bone metastasis of lung cancer cells. The interaction between tumor cells and the microenvironment is implicated in the malignant progression and growth of tumor cells [30]. In the current study, we demonstrated that lumican was overexpressed in lung cancer cells exhibiting a higher capacity for bone metastasis. Cell adhesion to the ECM and the development of bone metastasis was decreased after downregulation of lumican expression, suggesting that lumican promotes bone metastasis by modulating the interaction between tumor cells and the ECM.

Lumican, first identified as a major proteoglycan with a 38-KDa core protein in the cornea, is expressed in the ECM of several tissues [28,31]. The role of lumican in tumor progression remains unclear due to the conflicting results reported previously. The expression of lumican in melanomas inhibits cell invasion and tumor formation [24], whereas, in patients with lung adenocarcinoma, the level of lumican expression in the cancer cells is correlated with cell metastasis and tumor growth, but not with disease prognosis [32]. In breast cancer, the expression of lumican in cancer cells is correlated with young age, high-grade tumor, and low expression of the estrogen receptor [33]. In prostate cancer, the inhibitory role of lumican was supported by several studies [34,35]. Although the effects of lumican overexpression in different cancers were contradictory, the involvement of lumican in the regulation of tumor growth, cell mobility, and ECM attachment was demonstrated clearly.

The effect of lumican on the regulation of the collagen fibril assembly was well described [36]. In lumican-deficient mice, reduced tensile strength of the skin and loss of corneal clarity were observed [37]. In the bones, type I collagen is a major component of the organic part of the bone matrix and is secreted by differentiated osteoblasts [10,38]. Enhanced expression of lumican is observed at the stage of differentiation and mineralization in the osteoblasts, suggesting that lumican may be participating in the regulation of bone formation [10]. The study conducted to investigate the regulation of lumican in type I collagen fibrillogenesis demonstrated that the concentration of lumican determines the spacing and connectivity of collagen fibrils [39]. Additionally, in vitro collagen fibrillogenesis is completely abolished in the presence of a high concentration of lumican [39]. In this study, we demonstrated that the proteolytic activity associated with ECM degradation in the LLC/luc cells is elevated after lumican stimulation. These results suggested that the osteotropic lung cancer cells may affect the composition of the bone marrow microenvironment by secreting the lumican protein abundantly, resulting in an environment favoring the growth of cancer cells in the bones.

The role of lumican on tumor progression may be divided into several parts. A previous study [16] showed that overexpression of lumican enhanced the migration of colon cancer cells through modulating the organization of actin filaments, which supports our results in this study. A decreased migration and invasion ability was observed in osteotropic lung cancer cell lines transfected with lumican-specific shRNAs. Other studies demonstrated that soluble lumican protein deposits on the surface of neutrophils and promotes the recruitment of neutrophils to the inflammatory region [18,19], suggesting that lumican may also play a role in intravasation and extravasation. In addition, lumican, secreted by differentiating and mature osteoblasts, is a major component of bone matrix [10]. During the process of bone remodeling, the ECM components are released into bone microenvironment and create a concentration gradient of ECM proteins, including lumican, which may act as a chemoattractant to induce the migration of tumor cells in bone marrow. Therefore, soluble lumican, secreted by tumor cells or released from degraded bone matrix, may induce the cytoskeleton rearrangement of tumor cells through binding to self-expressed integrin receptor, which increases cell mobility and enhances the tumor adhering with bone matrix.

The core protein of lumican is expected to be around 37 kDa. Previous studies demonstrated that the molecular weight of lumican in lung cancer is about 37–100 kDa [32], indicating the presence of multiple forms of glycosylated lumican. The anti-lumican antibody (Ab) used in our study mainly recognizes a single band around 55 kDa (Figures S2, S4–S7, Supplementary Materials). After a long exposure time, the signals from lumican with molecular weights of 34–43 kDa and 95 kDa were observed in the Western blot (Figure S5, Supplementary Materials). These results indicated that the LLC and A549 cells mainly express a lightly glycosylated lumican with a molecular weight of 55 kDa, with a small amount of lumican with molecular weights of 34–43 kDa (likely to be the core lumican protein) and 95 kDa (likely to be a heavily glycosylated lumican). The lumican glycosylation pattern may affect its abilities to regulate various cellular functions. However, the molecular weight of the recombinant lumican protein we purchased from R&D was 55–65 kDa (Figure S3, Supplementary Materials). We showed that this 55–65-kDa recombinant lumican can exert the biological activity of lumican, such as inducing FAK phosphorylation and promoting cell adhesion. Therefore, these results suggest that the 55-kDa lumican expressed in LLC and A549 cells is likely to be responsible for the biological effects observed with the recombinant lumican. We cannot rule out the possibility that the 34–43-kDa and ~95-kDa forms of lumican may exert different biological effects both in vitro and in vivo; however, we believe that the 55-kDa lumican expressed in LLC and A549 cells is involved in the functions we observed in this study.

The parental LLC cells pre-incubated with lumican displayed an early seeding of cancer cells in the bone in mice after I.C injection (Figure 3F), suggesting that incubation with lumican increases the tumor bone metastasis capacity of the parental LLC cells. In our experimental setting, I.V. injection of the parental LLC cells resulted in artificial lung metastasis in 100% of mice, and no bone metastasis was observed before the death of mice due to outgrowth of tumor in the lung. However, I.V. injection of the selected, highly bone-tropic LLC tumor cells resulted in bone metastasis in 20% of mice, further indicating the enhanced bone metastasis capacity of the bone-tropic tumor cells. The luminescent signal in the abdominal region of mice in Figure 1A resulted from the tumor deposits in mesenteric lymph nodes. The appearance rate of luminescent signal in this region was less than 20%, and sometimes the signal vanished during the development of tumor bone metastasis. Tumor metastasis to other organs, such as spleen or liver, was observed in mice after I.C injection of the selected, highly bone-tropic LLC tumor cells. However, the rate of tumor metastasis to other organs was low as compared to bone (100%) in mice after I.C injection of the selected, highly bone-tropic LLC tumor cells. Therefore, we believe that the highly bone-tropic tumor cell line selected in our system displays a higher tropism to the bone.

The dominant role of the stromal cell-derived factor 1/C-X-C chemokine receptor type 4 (SDF-1/CXCR4) signaling pathway in promoting breast cancer-associated bone metastasis was described in several studies [40,41]. However, similar levels of SDF-1 and CXCR4 expression between parental and osteotropic LLC/luc cells were observed in our microarray analysis, suggesting that this pathway is not involved. Soluble lumican promotes the migration of neutrophils toward the inflammatory region by interacting with surface-expressed integrin [18,19]. Overexpression of lumican in colon cancer cells enhanced the cell motility through modulating the rearrangement of the actin cytoskeleton, and another study showed that lumican induced the migration of corneal epithelial cell via the ERK1/2 signaling pathway [16,20]. In contrast, co-culturing with exogenous lumican restored the activation of FAK in lumican knockdown cells and promoted the seeding of parental LLC/luc cells into the bone marrow. Collectively, the results in this study support the notion that lumican in the osteotropic LLC/luc cells promotes bone metastasis by modulating the interaction of cancer cells with the bone microenvironment and enhancing the settlement of cancer cells in the bone via an autocrine regulatory mechanism.

## 4. Materials and Methods

### 4.1. Animals and Cell Lines

All animal experimental procedures were approved (NHRI-IACUC-100145-A, NHRI-IACUC-100114-A) by the Institutional Animal Care and Use Committee of the National Health Research Institutes. Male C57BL/6J Narl (C57BL/6) and NOD-SCID mice aged 5–6 weeks were purchased from the National Laboratory Animal Center, Taiwan, and maintained in the animal facility of the National Health Research Institutes. The *luciferase*-transfected murine LL/2 Lewis lung carcinoma cells (LLC/luc) and human lung cancer cells (A549/luc) were purchased from Caliper Life Sciences (Alameda, CA, USA) in 2008. These cell lines were authenticated at Bioresource Collection and Research Center (BCRC, ROC) by analysis of 16 short tandem repeat loci on 28 August 2019. The LLC/luc cell line was maintained in Dulbecco’s modified Eagle medium (DMEM) (GE, Boston, MA, USA) supplemented with 5% fetal bovine serum (FBS) (Invitrogen, Carlsbad, CA, USA), and the A549/luc cell line was maintained in Roswell Park Memorial Institute (RPMI)1640 medium (GE) with 10% FBS. The bone metastatic LLC/luc cells (LLC/luc BM 2nd) and A549/luc cells (A549/luc BM 2nd) were generated from metastatic lesions of the bone as described previously [23].

### 4.2. Antibodies and Recombinant Proteins

Rabbit anti-lumican monoclonal antibody was purchased from Abcam (Cambridge, MA, USA). Mouse anti-actin monoclonal antibody was purchased from Millipore (Billerica, MA, USA). Horseradish peroxidase (HRP)-conjugated goat anti-rabbit immunoglobulin G (IgG) and HRP-conjugated goat anti-mouse IgG were purchased from Santa Cruz Biotechnology (Santa Cruz, CA, USA). Recombinant mouse lumican protein was purchased from Abcam.

### 4.3. Cell Proliferation Assay

The in vitro cell proliferation assay was performed as described previously [23]. Briefly, bone metastatic A549/luc (1 × 10^5^ cells/well) and LLC/luc (5 × 10^4^ cells/well) cells transfected with a control plasmid and a lumican-specific shRNA plasmid were seeded onto a 24-well culture plate (Nunc, Life Technologies). After incubating for 0, 24, 48, and 72 h at 37 °C, the cell number was determined using a tetrazolium-based assay (MTT assay) (Sigma-Aldrich, St. Louis, MO, USA).

### 4.4. Cell Adhesion Assay

For the cell adhesion assay, a 96-well plate was coated with several ECM components including fibronectin, collagen type I, collagen type IV, and laminin (10 μg/mL, 100 μL/well) (Sigma-Aldrich) and incubated for 30 min at 37 °C. After washing with phosphate-buffered saline (PBS), the ECM component-coated wells were blocked with 3% bovine serum albumin (BSA in PBS) for 2 h at room temperature. After washing with PBS, the control plasmid and the lumican-specific shRNA plasmid-transfected bone metastatic A549/luc and LLC/luc cells were overlaid onto the ECM component-coated surfaces (2 × 10^4^ cells/well), and the plate was incubated for 1 h at 37 °C. Upon removing the non-adherent cells by washing with PBS, calcium-AM (100 μM in ethylenediaminetetraacetic acid/PBS, 100 μL/well) (Thermo Fisher Scientific, Waltham, MA, USA) was added to the wells, and the plate was incubated for another 30 min at 37 °C. The cell suspensions were transferred to a Costar^TM^ 96-well black plate (Thermo Fisher Scientific), and the fluorescence (excitatiion/emission = 485/535 nm) was measured using a spectrophotometer (Spectra Max M5; Molecular Devices, Sunnyvale, CA, USA). To evaluate the effect of exogenous recombinant lumican protein on the adhesion of cancer cells, lumican knockdown LLC/luc BM 2nd cells, co-cultured with and without lumican protein, were overlaid onto the ECM component-coated surfaces (2 × 10^4^ cells/well), and the cell adhesion was determined, as described above.

### 4.5. Migration and Invasion Assay

The in vitro cell migration assay was performed using a Transwell culture insert (8 μm, Corning, Tewksbury, MA, USA). The control plasmid and lumican-specific shRNA plasmid-transfected bone metastatic A549/luc and LLC/luc cells were suspended in serum-free RPMI-1640 medium and DMEM, respectively, and seeded onto the culture insert. Then, the culture insert was placed into a well containing complete medium with 5% FBS as a chemoattractant and cultured in a CO_2_ incubator at 37 °C for 24 h. After incubation, the culture insert was removed, and cells that remained in the culture insert were scraped off. The cells that migrated through the pores and attached themselves to the lower side of culture insert were fixed in methanol, stained with Giemsa solution (Sigma-Aldrich), and counted under a microscope to determine the extent of cell migration. The in vitro cell invasion assay was performed by following the methods described above but using the BioCoat^TM^ Matrigel^®^ Invasion Chambers (Corning) as the culture insert instead of the Transwell culture insert. To evaluate the effect of exogenous recombinant lumican protein on the invasion capacity of cancer cells, lumican knockdown LLC/luc BM 2nd cells, co-cultured with and without the lumican protein, were used to perform the in vitro cell invasion analysis by following the methods described above.

### 4.6. shRNA Transfection

All shRNA plasmids were purchased from the RNAi core of the Academia Sinica (Nankang, Taiwan). Target sequences of the shRNA plasmid were as follows: 5′-CCGGCCTGGAAACTCGTTTAATATACTCGAGTATATTAAACGAGTTTCCAGGTTTTTG-3′ and 5′-CCGGCGATTATGACATCCCTCTCTTCTCGAGAAGAGAGGGATGTCATAATCGTTTTTG-3′ for mouse lumican (NM_008524); 5′-CCGGGCATTGCAGTATCTGCGTTTACTCGAGTAAACGCAGATACTGCAATGCTTTTTTG-3′ and 5′-CCGGCCAGAATGTAACTGCCCTGAACTCGAGTTCAGGGCAGTTACATTCTGGTTTTTTG-3′ for human lumican (NM_002345); 5′-CCGGCAACAGCCACAACGTCTATATCTCGAGATATAGACGTTGTGGCTGTTGTTTTTG-3′ for the control shRNA. The transfection of shRNA into cancer cells was performed by following the manufacturer’s instructions on the TurboFect Transfection Reagent (Thermo Fisher Scientific).

### 4.7. Western Blotting

Cells were lysed in a lysis buffer containing protease inhibitor (Sigma-Aldrich). The expression of lumican was detected using rabbit anti-lumican (1:5000), and actin was used as an internal control.

### 4.8. RNA Isolation and Quantification

Total RNA was extracted by following the manufacturer’s protocol of the RNeasy Mini Kit (QIAGEN, Valencia, CA, USA), and the complementary DNA (cDNA) was synthesized by following the manufacturer’s protocol of the SuperScript^TM^ III First-Strand Synthesis SuperMix for qRT-PCR (Thermo Fisher Scientific). The gene expression of lumican was determined by following the manufacturer’s protocol of the SYBR^®^ Green PCR Master Mix Kit (Applied Biosystems, Thermo Fisher Scientific). The primer sequences that were used are as follows: 5′-CATTAGTCGGTAGTGTCAGTG-3′ and 5′-TGCCAGGAGGAACCATTG-3′ for mouse lumican; 5′-CTGAGAGGGAAATCGTGCG-3′ and 5′-GGTGGTACCACCAGACAGC-3′ for mouse beta-actin; 5′-TGCCCTGAAAGCTACCCAAG-3′ and 5′-TGAGCCACTGCAGATCAGTT-3′ for human lumican; 5′-CTCTGCTCCTCCTGTTCGAC-3′ and 5′-GCGCCCAATACGACCAAATC-3′ for human GAPDH. The gene expression of lumican was analyzed using the 7500 Fast &7500 Real-Time PCR System (Applied Biosystems, Thermo Fisher Scientific).

### 4.9. In Vivo Animal Model

The control plasmid and the lumican-specific shRNA plasmid-transfected bone metastatic A549/luc and LLC/luc cells (1 × 10^5^ cells/mouse) were injected I.V. and I.C. for the establishment of lung and bone metastases, respectively, as described previously [23]. Colonization of the bone and lung metastases was observed using the IVIS^TM^ live-imaging system (IVIS Lumina II, PerkinElmer, SantaClara, CA, USA). To assess the autocrine effect of lumican on the establishment of bone metastasis of lung cancer cells, bone metastatic or parental LLC/luc cells, cultured with and without recombinant lumican (100 ng/mL) at 37 °C for 24 h, were I.C. injected into mice (1 × 10^6^ cells/mouse). The total bone marrow cells were harvested from the mice one day after the I.C. injection, and the cDNA was synthesized as described above. The gene expression for luciferase was determined by real-time PCR following the manufacturer’s protocol of the SYBR^®^ Green PCR Master Mix Kit. The primer sequences that were used are as follows: 5′-GCTGGGCGTTAATCAGAGAG-3′ and 5′-GTCGAAGATGTTGGGGTGTT-3′ for firefly luciferase; 5′-TTCCAGTATGACTCCACTCA-3′ and 5′-ATCACGCCACAGCTTTCCAG-3′ for mouse GAPDH. The gene expression for luciferase was analyzed using a 7500 Fast Real-Time PCR System and the identity of products obtained after the PCR reaction was confirmed by agarose gel electrophoresis.

### 4.10. MMP2/9 Activity Assay

Lumican knockdown bone metastatic LLC/luc cells were cultured in a serum-free medium containing 10 ng/mL and 100 ng/mL recombinant lumican protein at 37 °C. In total, 100 μL of the culture supernatant was collected after intervals of 30 min and incubated with Oregon Green^®^ 488-conjugated gelatin (Molecular Probes, Thermo Fisher Scientific) as a substrate for MMP2/9. The increased intensity of fluorescence was measured using a Spectra Max M5 spectrophotometer.

### 4.11. Lumican ELISA

To determine the level of lumican in the culture supernatant, the parental and bone metastatic LLC/luc cells suspended in complete medium were seeded onto 10-cm culture plates (2 × 10^6^ cells/plate) and were incubated at 37 °C for 6 h. The complete medium was replaced with serum-free medium after washing with PBS, and the plates were incubated at 37 °C for 24 h. The culture supernatant was collected and filtered with a 0.22-μm filter to remove cell debris. The level of tumor-secreted lumican in the culture supernatant was determined following the manufacturer’s protocol of the Lum (mouse) ELISA kit (Abnova, Taipei, Taiwan).

### 4.12. Statistical Analysis

All data are presented as means ± SEM. The Student’s *t*-test and the log-rank test were used to analyze the data with the GraphPad Prism 4.0 software (GraphPad Software, Inc., La Jolla, CA, USA). A *p*-value ≤ 0.05 was considered statistically significant (* *p* < 0.05, ** *p* < 0.01, and *** *p* < 0.001).

## 5. Conclusions

Our findings indicate that lumican modulates the interaction between lung cancer cells and bone microenvironment and promotes the settlement of cancer cells in the bone through an autocrine regulatory mechanism.

## Data Availability

The microarray data analyzed in this study were deposited at the Gene Expression Omnibus (GEO) database with accession number GSE131614. Data and materials are available from the corresponding author on reasonable request.

## Supplementary Materials

The following are available online at https://www.mdpi.com/2072-6694/12/1/233/s1, Figure S1. The expression of integrin β1 and β2 in the surface of bone metastatic LLC/luc cells. The surface expression of integrin β1(left panel) and β2 (right panel) in bone metastatic LLC/luc cells was determined by flow cytometric analysis. Key: ━: Isotype; --: β1 or β2 specific antibody, Figure S2. Exogenous lumican induced the FAK phosphorylation in LLC/luc cells, Figure S3: Detection of the recombinant mouse lumican protein with anti-lumican Ab. The recombinant mouse lumican protein (1 μg/lane) was detected with a rabbit anti-lumican Ab, Figure S4: Full blots corresponding to Figure 1, Figure S5: Full blots corresponding to Figure 2, Figure S6: Full blots corresponding to Figure 4, Figure S7: Full blots corresponding to Figure 5, Table S1: List of the top 10 up-regulated genes in bone metastatic LLC/luc cells, Table S2: Upregulation of genes associated with ECM and EMT in bone metastatic LLC/luc cells.
