# Supplementary material for: Elevated Expression of Lumican in Lung Cancer Cells Promotes Bone Metastasis through an Autocrine Regulatory Mechanism
Source: Cancers (Basel). 2020 Jan 17;12(1):233. doi: 10.3390/cancers12010233 (PMC7016828; doi:10.3390/cancers12010233)
Supplement: Supplementary file 1 [file cancers-12-00233-s001.pdf]

Supplemental Materials: Elevated Expression of Lumican in Lung Cancer Cells Promotes Bone Metastasis through an Autocrine Regulatory Mechanism

Kuan-Chung Hsiao, Pei-Yi Chu, Gee-Chen Chang and Ko-Jiunn Liu

FAK activation: Cells were cultured in growth medium containing with or without recombinant lumican protein (100 ng/mL) for 8 h. The cell lysates were harvested and the protein expression was determined by Western blotting analysis. The expression level of detected proteins in each cell was normalized to internal control (actin) individually and the numbers indicated that the expression level of FAK, pFAK and lumican in cells under different treatment as compared to that in the cells transfected with control vector (VC).

Integrin expression: For examination of surface staining of integrin 1 and 2 in osteotropic LLC/luc lung cancer cells, cells were washed with PBS and then stained with 1 g of the corresponding Ab at 4 °C for 1 h. After washing with PBS, the cells were stained with PE-conjugated goat anti-mouse IgG at a dilution of 1:200 at 4 °C for 1 h. After further washing with PBS, the cells were suspended in PBS and the expression of integrin 1 and 2 on the surface of osteotropic LLC/luc cells were analyzed by a flow cytometer (FACS Calibur; Becton Dickinson, Franklin Lakes, NJ, USA).

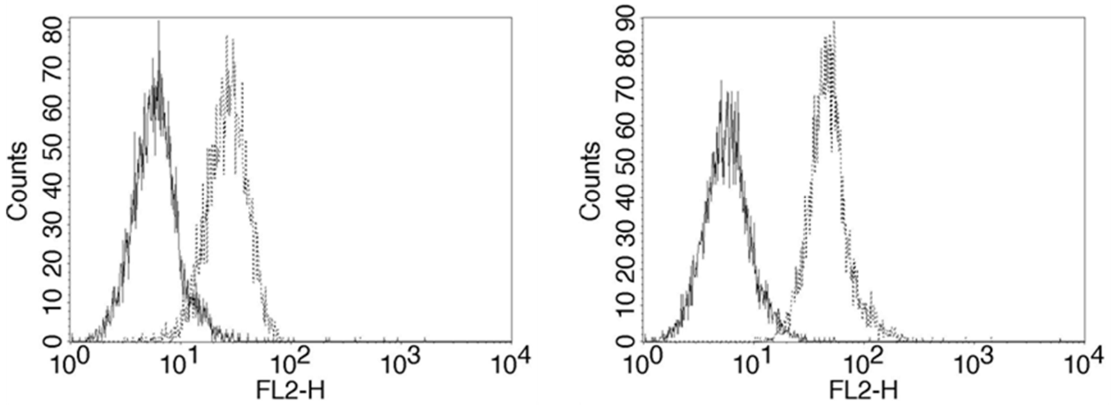

Figure S1. The expression of integrin 1 and 2 in the surface of bone metastatic LLC/luc cells. The surface expression of integrin 1(left panel) and 2 (right panel) in bone metastatic LLC/luc cells was determined by flow cytometric analysis. Key: —: Isotype; --: 1 or 2 specific antibody.

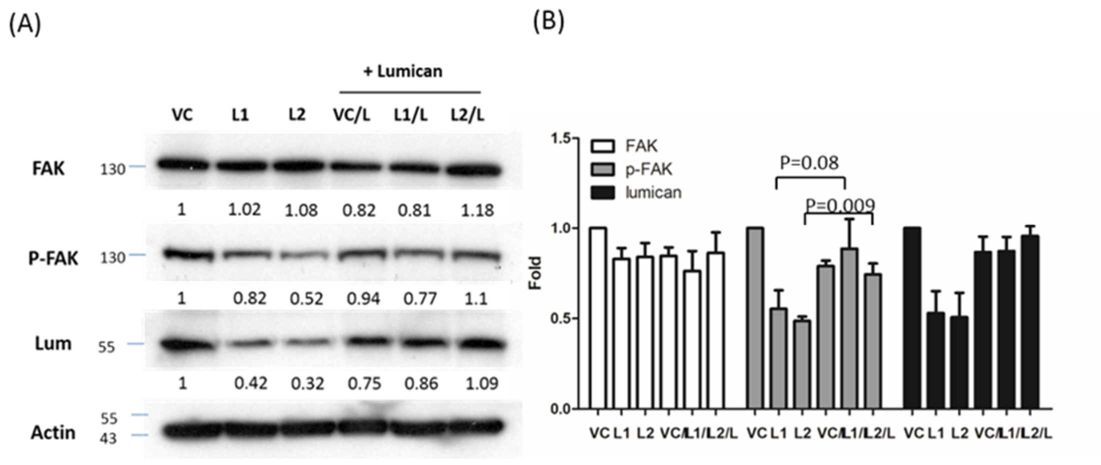

Figure S2. Exogenous lumican induced the FAK phosphorylation in LLC/luc cells. (**A**) The expression of FAK, p-FAK 397 and lumican (Lum) was determined in LLC/luc BM 2nd cells transfected with a control vector (VC) and a lumican-specific shRNA plasmid (L1 and L2) after incubation with (+lumican) and without the recombinant lumican protein. The levels of FAK, p-FAK 397 and lumican expression in each cell was individually normalized to the internal control (actin) and the numbers indicated the level of FAK, p-FAK 397 and lumican expression in lumican-knockdown bone metastatic LLC/luc cells as compared to that in the cells transfected with a control vector (**B**). The levels of FAK, p-FAK 397 and lumican expression in each cell stimulated with and without the recombinant lumican protein from three independent experiments were quantified.

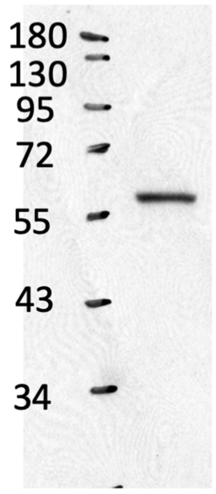

Figure S3. Detect the recombinant mouse lumican protein with anti-lumican Ab. The recombinant mouse lumican protein (1 μg/lane) was detected with rabbit anti-lumican Ab.

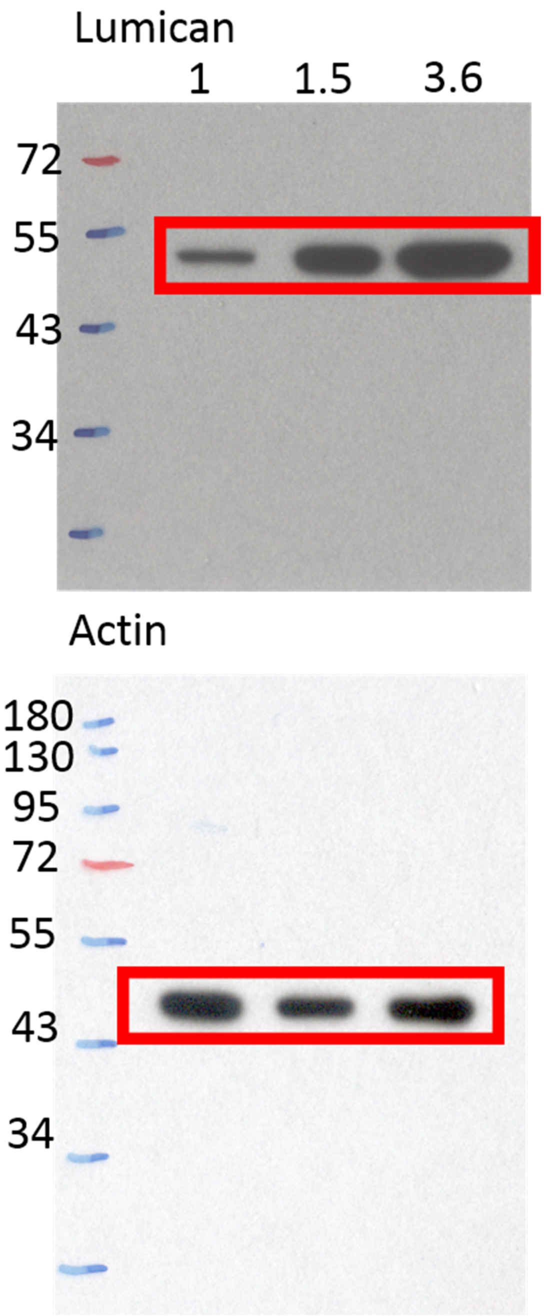

Figure S4. Full blots corresponding to Figure 1.

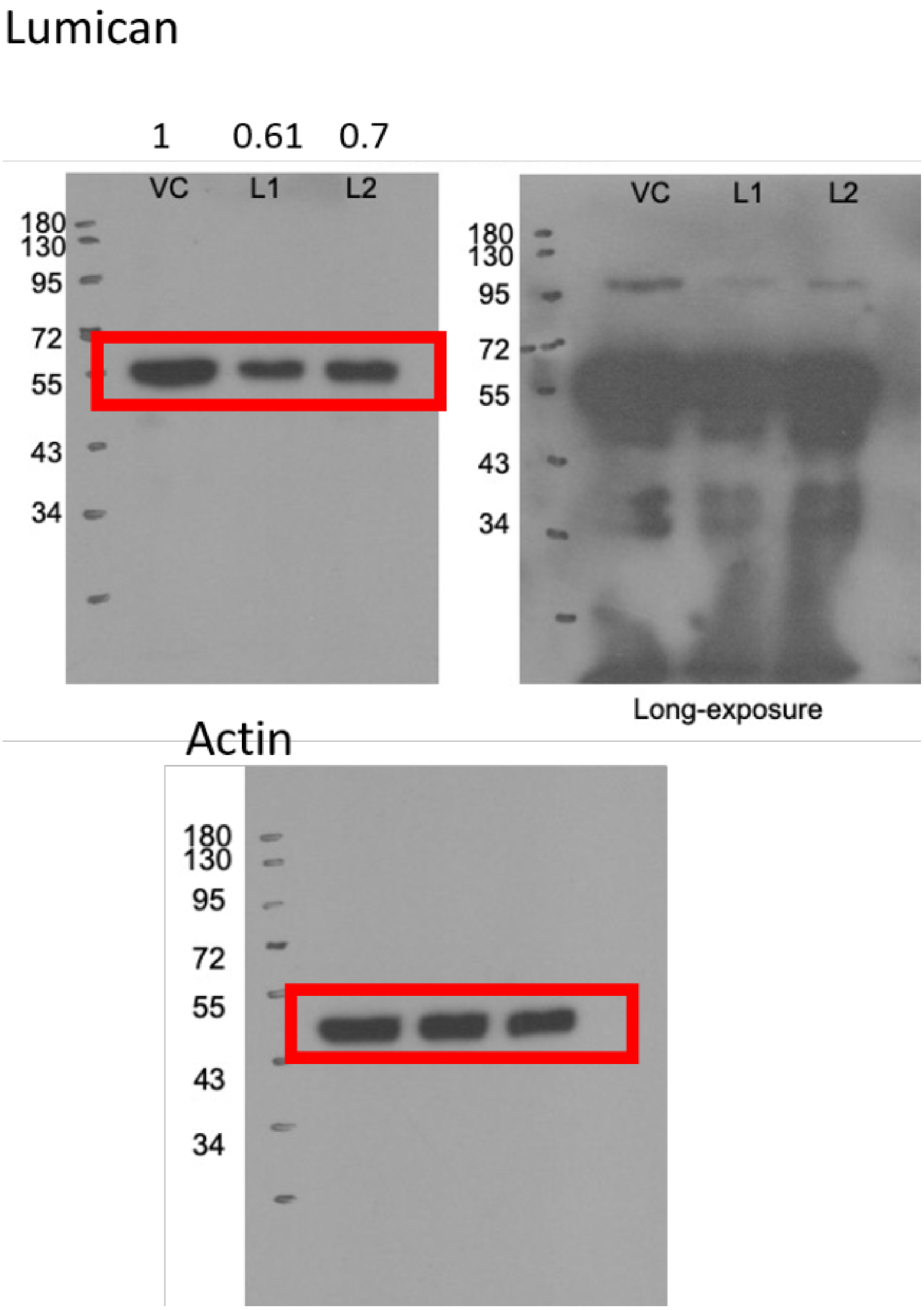

Figure S5. Full blots corresponding to Figure 2.

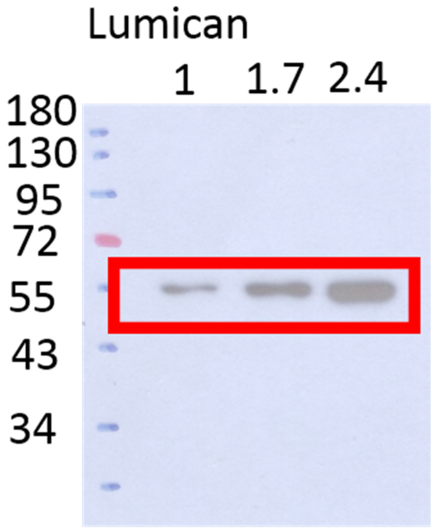

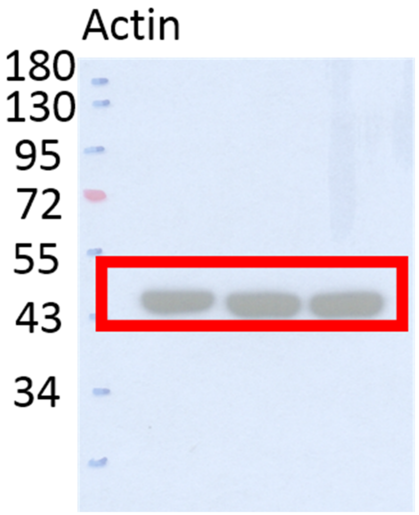

Figure S6. Full blots corresponding to Figure 4.

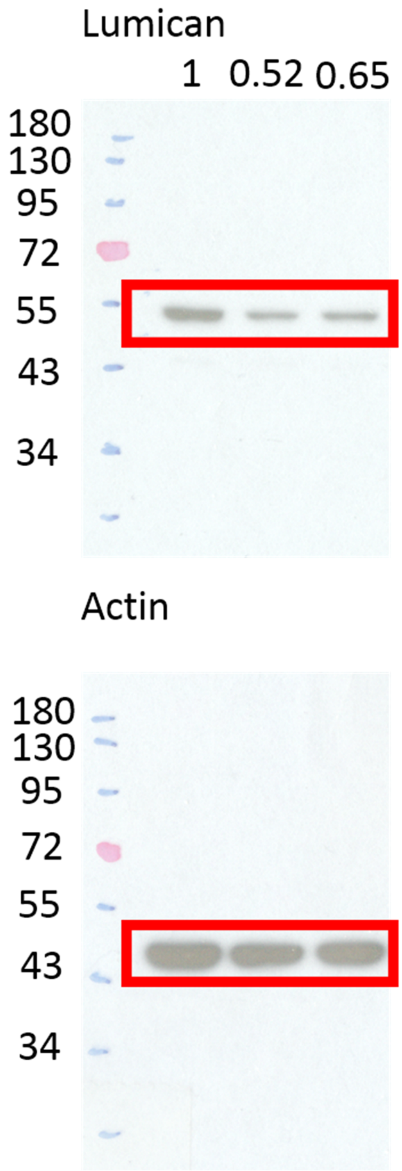

Figure S7. Full blots corresponding to Figure 5.

Table 1. List of the top 10 up-regulated genes in bone metastatic LLC/luc cells. The significant, differentially expressed genes were identified as |log2 (Genes expressed in LLC BM^{2nd}/Genes expressed in LLC P)|>1, and the gene encoding for lumican, *LUM*, displayed the highest fold change of differentially expressed gene.

Genes	Log2 BM 2nd/P
LUM	4.953367132
CCL7	4.562387198
AQP1	4.432948185
MGP	4.222222089
COL6A3	4.176850604
ARHGDIB	4.136196053
UGT1A10	4.051044574
DPT	3.965711237
LOXL1	3.912371302
IRF1	3.69191869

Table 2. Upregulation of genes associated with ECM and EMT in bone metastatic LLC/luc cells. The significant differentially-expressed genes were identified as |log2 (Genes expressed in LLC BM2nd/Genes expressed in LLC P)|>1, and 269 genes were up-regulated in LLC BM 2nd cells. We found that lumican displayed the highest fold change of differentially-expressed gene. After analyzing the functional enrichment of differentially-expressed genes by using Database for Annotation, Visualization, and Integrated Discovery (DAVID) online tool, the functional annotation results showed that the genes associated with extracellular matrix, including several EMT associated genes, such as, *AEBP1*, *S100A4*, *FGF10*, *LUM*, *NRP1* and *TNC*, were the most enriched in our model. Besides those EMT-associated genes, several genes encoding for ECM associated protein were enriched in this model, such as CCL7, COL6A3, DCN, DPT, ECM1, LUM and MGP.

Genes	Log2 BM 2nd/P
LUM	4.953367132
CCL7	4.562387198
MGP	4.222222089
COL6A3	4.176850604
DPT	3.965711237
DCN	2.588325941
CCL2	2.390504203
ECM1	1.042560112
FGF10	1.655279042
AEBP1	1.353184152
NRP1	1.325302449
S100A4	1.244187087
TNC	1.161691818

© 2020 by the authors. Licensee MDPI, Basel, Switzerland. This article is an open access article distributed under the terms and conditions of the Creative Commons Attribution (CC BY) license (http://creativecommons.org/licenses/by/4.0/).
